# Benzoate of Soda in Parasitic Diseases

**Published:** 1880-07

**Authors:** 


Benzoate of Soda in Parasitic Diseases.—The substance is greatly vaunted in Germany as a specific against parasitic diseases, among which are ranked pneumonia and pulmonary tuberculosis. Rokitansky makes his patients inhale, by means of a spray apparatus, a gram of the drug for each kilogram of bodily weight. Schwitzler, also partisan of the anti-parasitic treatment of phthisis, gives preference to inhalations and sub-cutaneous injections of phenic acid. Klebs, of Prague, signals the efficacy of benzoate of soda in all febrile diseases having an infectious character. The fever does not yield as quickly as with quinine or salicylate of soda, but it disappears in a more certain and permanent manner. Letzerich recommends it in the treatment of diphtheria. Upon twenty-seven patients treated during an epidemic he affirms to have lost but one, and that a child. In these cases the benzoate of soda is employed internally, the dose being from five to twenty grams, according to age, in about six ounces of vehicle, and externally in powder applied to the affected parts.

